# Unified Cross-Variable Correlation Learning for Long-Term Multivariate Time Series Forecasting

**DOI:** 10.3390/s26196238

**Published:** 2026-10-01

**Authors:** Fengrong Tang, Qinghui Wang, Jiang Chang, Huaiyu Xu, Chun Zheng, Zhihua Chen

**Affiliations:** 1 National Key Laboratory of Transient Physics, Nanjing University of Science and Technology, Nanjing 210094, China; tfr0427@njust.edu.cn (F.T.);; 2 School of Information Engineering, Yancheng Institute of Technology, Yancheng 224051, China; 3 College of Carbon Neutrality Future Technology, Sichuan University, Chengdu 610207, China; 4 School of Energy and Power Engineering, Nanjing University of Science and Technology, Nanjing 210094, China; 5 National Graduate College for Engineers, Southern University of Science and Technology, Shenzhen 518055, China

**Keywords:** time series forecasting, cross-variable correlation, sparse dependency modeling

## Abstract

The complex evolution of multivariate time series is jointly shaped by the temporal variability, heterogeneity, and selectivity of cross-variable correlations. However, existing forecasting methods typically focus on only one or a subset of these properties, potentially propagating temporally misaligned, polarity-conflicting, or irrelevant information across variables and thereby limiting forecasting performance. To address this issue, we propose TimeCorr, a unified correlation learning framework for multivariate time series forecasting that jointly models dynamic correlation, heterogeneous correlation, and selective correlation. Specifically, we first propose a Dynamic Correlation Module that estimates signed cross-variable correlation weights at different temporal positions to characterize temporal variations in dependency strength. On this basis, we design a Heterogeneous Correlation Module that independently calibrates the positive and negative components of dynamic correlation weights to capture polarity-specific interaction effects. Furthermore, we construct a Selective Correlation Module that learns sparse relation masks to filter the calibrated correlation weights, retaining informative variable pairs while suppressing irrelevant interactions. Finally, the sparsified correlation weights are used to aggregate local contextual information across variables for forecasting. Extensive evaluations on eight benchmark datasets demonstrate the consistently superior forecasting performance of TimeCorr, which achieves the lowest errors in 55 evaluation cases. Relative to the second-best results in the corresponding settings, TimeCorr reduces MSE and MAE by up to 6.12% and 4.57%, respectively.

## 1. Introduction

Multivariate time series are ubiquitous in modern sensor-enabled systems, including intelligent transportation [1], smart manufacturing [2], and healthcare [3]. Continuously collected from multiple sensors or data channels, these time-ordered observations provide a dynamic representation of system states and evolution. They encode not only recurring temporal patterns but also interactions among system components. Accurately forecasting these time series enables system operators to anticipate demand variations, identify emerging risks, and implement interventions before performance degradation, facilitating a transition from reactive response to proactive management. However, multivariate time series often exhibit intricate cross-variable dependencies [4], posing substantial challenges to reliable forecasting.

Multivariate time series forecasting aims to leverage historical observations to estimate the future trajectories of multiple correlated variables. In particular, long-term multivariate time series forecasting (LMTSF) extends the prediction horizon beyond near-future observations, providing broader temporal information for applications that require advance planning, resource allocation, and sustained decision support. Existing approaches to LMTSF can be broadly categorized into traditional statistical methods and deep learning-based methods. Traditional methods, such as ARIMA [5] and VAR [6], typically rely on predefined statistical assumptions and are effective in modeling relatively simple temporal structures. In contrast, deep learning-based methods [7] offer greater flexibility in representing nonlinear dynamics and high-dimensional dependencies through hierarchical feature learning, making them more suitable for complex multivariate time series.

Among deep learning-based approaches, considerable efforts have been devoted to modeling temporal dependencies, with convolutional, recurrent, and attention-based architectures being widely adopted to capture temporal patterns at different scales [8]. Meanwhile, temporal modeling alone is insufficient for multivariate time series, where variables often evolve jointly and exhibit complex interactions with one another [9]. Rather than being static and homogeneous, such dependencies often exhibit distinct structural properties across variable pairs and temporal contexts. Consequently, cross-variable dependency modeling has emerged as an important research direction for exploiting informative cross-variable interactions.

Recent studies have highlighted three complementary characteristics of inter-variable correlations. Dynamic correlation (DCorr) characterizes the temporal variation in inter-variable correlations, indicating that the correlation strength between the same pair of variables may change across different temporal contexts [10]. Heterogeneous correlation (HCorr) characterizes the polarity of inter-variable dependencies by distinguishing positive and negative correlation [11]. Selective correlation (SCorr) reflects the selectivity of inter-variable correlations, indicating that informative correlations may exist only among a subset of variable pairs, whereas indiscriminately modeling all pairwise interactions may introduce redundant information and noise [12,13,14]. These correlation characteristics have been explored from different perspectives. Crossformer [4] implicitly models DCorr through segment-wise cross-dimensional interactions, but does not explicitly account for HCorr or SCorr. CrossGNN [11] explicitly models HCorr using cross-variable graph structures with positive and negative edge weights, while partially addressing SCorr, but does not explicitly model DCorr. SageFormer [15] primarily addresses SCorr by learning a sparse directed graph and retaining only the Top-*K* neighbors of each variable, whereas its globally shared non-negative adjacency matrix does not explicitly model DCorr or HCorr. Despite these advances, the aforementioned methods address these correlation characteristics either in isolation or only in partial combinations, lacking a unified mechanism for jointly modeling DCorr, HCorr, and SCorr. Consequently, outdated relationships, sign-conflicting signals, or irrelevant information may be propagated to the target variable, ultimately contaminating its representation and degrading forecasting performance.

To bridge this gap, we propose TimeCorr, a unified inter-variable correlation learning framework that jointly characterizes the temporal variability, polarity heterogeneity, and interaction selectivity of inter-variable dependencies. By progressively refining correlation weights within an end-to-end forecasting framework, TimeCorr provides a more comprehensive alternative to conventional single-structure correlation modeling. The main contributions of this work are summarized as follows:

- We design a temporally conditioned Dynamic Correlation Module that constructs position-specific signed correlation weights. This design enables fine-grained tracking of dependency-strength variations throughout the look-back window, avoiding the temporal rigidity imposed by globally shared correlation structures.
- We develop a polarity-disentangled Heterogeneous Correlation Module that decomposes dynamic correlation weights into positive and negative branches and calibrates them independently. This dual-branch mechanism preserves polarity-specific interaction semantics and prevents dependencies with opposing effects from being conflated during cross-variable information propagation.
- We introduce a Selective Correlation Module together with a Correlation-guided Aggregation Mechanism. The former learns a sparse relation mask to adaptively identify informative variable pairs. The latter uses the retained positive and negative correlation weights to guide cross-variable aggregation of local contextual features, producing correlation-aware representations for forecasting.
- Extensive experiments on eight benchmark datasets demonstrate the effectiveness of TimeCorr relative to representative forecasting baselines.

## 2. Related Work

### 2.1. Multivariate Time Series Forecasting

Multivariate time series forecasting models can generally be categorized into statistical and deep models. Traditional tools, such as VAR and VARMA [16], typically characterize inter-series dynamic relationships based on linear assumptions. However, the complex nonlinear dependencies in real-world data limit their effectiveness in practical applications. With the development of artificial intelligence, various deep models have been proposed, exhibiting superior empirical performance in capturing nonlinear patterns. Among them, recurrent neural networks (RNNs) and convolutional neural networks (CNNs) have shown strong performance in short-term time series forecasting tasks [17,18,19]. Nevertheless, confined by the maximum propagation length of signal paths, such models face difficulties in modeling long-term dependencies. Many studies indicate that attention-based frameworks offer substantial advantages in modeling long-term dependencies, thereby demonstrating significant potential for accurate LMTS [20,21].

### 2.2. Cross-Variable Dependency Learning for LMTSF

Cross-variable dependency learning has become an important research direction in LMTSF. This is particularly evident in industrial applications, where measurements from different process variables are coupled through the underlying operating process. AKGNN [22] adaptively constructs a graph over process variables for industrial soft sensing, while GraphSiamese [23] exploits graph-structured spatial relationships and slow temporal dynamics for process quality prediction. From a broader smart-manufacturing perspective, Arman et al. [24] demonstrate the integration of heterogeneous sensor information within an explicit production workflow. These studies highlight the practical importance of identifying and exploiting informative relationships among variables in multivariate systems.

Early studies generally model variable interactions through a unified correlation structure. MTGNN [25] represents variables as graph nodes and adaptively learns their connectivity for information propagation. iTransformer [26] applies self-attention across variable tokens to learn multivariate correlations, while their temporal representations are independently encoded by feed-forward networks. A2TFFNet [27] further addresses non-IID multivariate time series through adaptive two-dimensional time-frequency fusion, enhancing the representation of complex multivariate patterns and non-stationary abrupt changes. Chen et al. [28] further improve dependency representation by compressing the spatial dimension through low-rank embedding while extending the temporal context to capture longer-range dynamics, thereby achieving a better balance between spatial and temporal information. These methods effectively exploit multivariate information, they generally focus on unified feature without explicitly distinguishing different characteristics of the underlying inter-variable relationships.

In real-world multivariate time series, inter-variable relationships are considerably more complex and can be characterized from three complementary perspectives. For DCorr, SDGL [29] decomposes inter-variable dependencies into stable long-term and dynamic short-term components, with the latter represented by time-varying graphs inferred from local node features. MTGODE [30] models multivariate time series as dynamic graphs with evolving node states and learns their continuous spatiotemporal dynamics through graph neural ordinary differential equations. MAGNN [31] further infers scale-specific inter-variable dependency graphs to capture correlation patterns at different temporal resolutions. DADiffNet [32] models delayed spatiotemporal interactions through traffic-flow increments and adaptive local subgraphs, enabling dynamic selection of informative neighborhoods while capturing inter-node propagation delays.

For HCorr, TimeCNN [33] preserves both positive and negative interactions through unconstrained convolutional weights. CrossGNN [11] constructs signed interaction graphs that distinguish positive, negative, and neutral relationships during cross-variable message passing, while SEED [34] introduces a signed graph constructor to prevent negative dependencies from being eliminated or reversed by softmax normalization. These studies indicate that explicitly preserving correlation polarity can provide dependency information that is overlooked by non-negative attention or graph weights.

For SCorr, the transfer-entropy graph approach [35] restricts cross-variable propagation to directed dependencies supported by transfer entropy rather than assuming that every variable affects every other variable. DUET [14] combines channel soft clustering with graph sparsification to suppress noisy interactions. Adapformer [36] selectively incorporates essential cross-channel dependencies and focuses its forecasting stage on the most relevant covariates. TimeFilter [37] further performs patch-specific edge filtration, retaining only the dependencies relevant to the current temporal context. It has explored a closely related direction by modeling dynamic, heterogeneous, and selectively occurring cross-variable correlations for pretrained time-series foundation models. Its correlation modeling is mainly based on low-rank decomposition and contrastive representation learning, reflecting a carefully designed strategy for lightweight adaptation; however, it does not explicitly maintain a sparse signed interaction structure for direct cross-variable information propagation. TimeCorr, by comparison, constructs position-specific signed relation matrices, models positive and negative dependencies separately through HCorr, and employs Hard-Concrete $L_{0}$-regularized SCorr to adaptively suppress weak or redundant relations. The retained relations are subsequently incorporated into correlation-guided variable aggregation. Accordingly, the principal novelty of TimeCorr relative to CoRA lies in the explicit integration of dynamic relation estimation, polarity-specific dependency modeling, sparse relation selection, and relation-guided information propagation within the forecasting process. From an application perspective, CoRA is intended as a plug-and-play adaptation module for pretrained time-series foundation models, whereas TimeCorr is formulated as a standalone LMTSF framework for explicit cross-variable dependency modeling.

## 3. Methodology

### 3.1. Problem Definition

This study focuses on the task of long-term multivariate time series forecasting. Let $x_{t}\in R^{D\times L}$ denote the values of *D* sequences at time step *t*. Given a historical multivariate time series sample of length *L*, the goal of the long-term multivariate time series forecasting task is to predict the values of the next *H* steps. This paper aims to learn the mapping relationship between the input and the output through the proposed model.

### 3.2. Structure Overview

**Figure 1:**
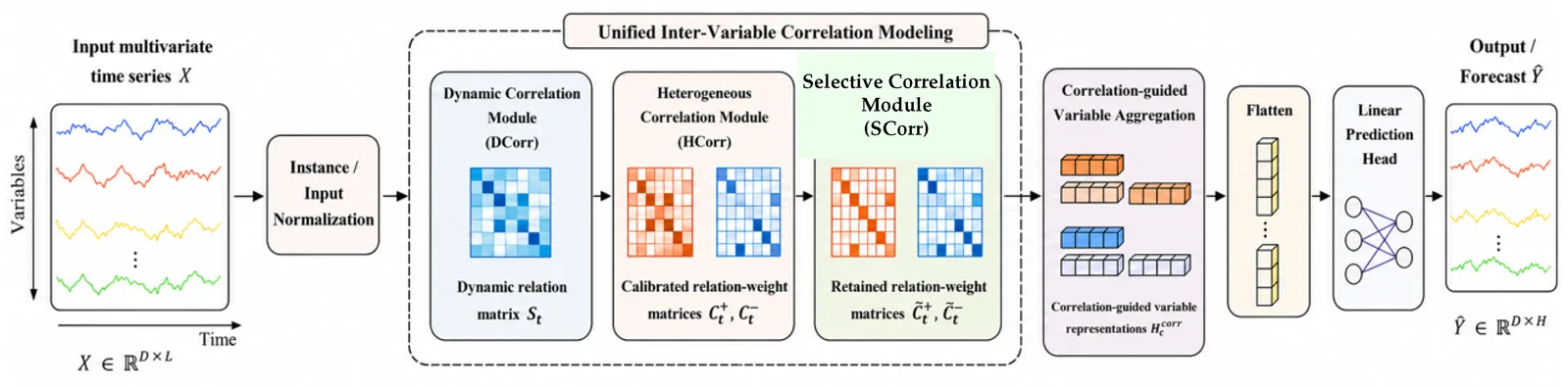
Overview of the proposed method.

The overall architecture of TimeCorr is illustrated in Figure 1. TimeCorr employs a progressive correlation-learning framework to jointly characterize three complementary properties of inter-variable dependencies: dynamic correlation (DCorr), heterogeneous correlation (HCorr), and selective correlation (SCorr). Specifically, the Dynamic Correlation Module independently estimates a signed matrix of inter-variable correlation weights at each temporal position, thereby capturing temporal variations in dependency strength. The Heterogeneous Correlation Module subsequently decomposes this matrix into positive and negative correlation-weight components and calibrates them independently to model the heterogeneous interaction effects associated with different correlation polarities. On this basis, the Selective Correlation Module applies an $L_{0}$-regularized stochastic gating mechanism to adaptively sparsify the correlation weights, retaining only those associated with informative variable pairs. The retained positive and negative correlation weights separately guide cross-variable feature aggregation, and the final aggregated representation is subsequently fed into a linear forecaster to generate forecasts. The model is jointly optimized using the forecasting loss and the sparsity regularization loss.

### 3.3. Dynamic Correlation Module

**Figure 2:**
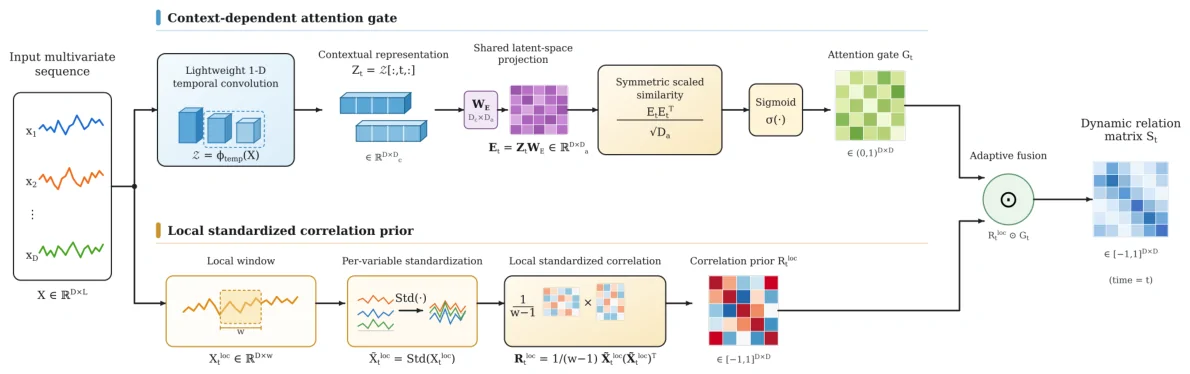
The structure of the Dynamic Correlation Module.

DCorr characterizes temporal variations in the strength and polarity of inter-variable dependencies, indicating that the same pair of variables may exhibit different correlation patterns under different temporal contexts. Attention-based cross-variable modeling provides a natural mechanism for capturing such variations, since pairwise interactions can be adaptively determined from context-dependent variable representations [4]. However, the sign of an unconstrained attention score does not necessarily correspond to the statistical correlation polarity of the original variables. To address this issue, we introduce a local standardized correlation matrix as a statistical prior and use a context-dependent attention gate to adaptively calibrate its contribution, as illustrated in Figure 2. Given an input multivariate time series

(1) $$X\in R^{D\times L}$$

where $X_{i,:}$ denotes the observations of the *i*-th variable over the look-back window, we first extract context-dependent representations using a lightweight temporal encoder:

(2) $$Z=\phi_{\mathrm{temp}}\left(X\right)\in R^{D\times L\times D_{c}}$$

where $\phi_{\mathrm{temp}}\left(\cdot \right)$ denotes a lightweight one-dimensional temporal convolutional operator and $D_{c}$ denotes its output dimension. The contextual representations of all variables at temporal position *t* are denoted as

(3) $$Z_{t}=Z\left[:,t,:\right]\in R^{D\times D_{c}}$$

The *i*-th row $z_{t,i}$ encodes the local temporal state of the *i*-th variable around temporal position *t*. Therefore, the subsequent inter-variable relation modeling is conditioned on the local temporal context rather than on isolated observations. To provide the learned relations with explicit statistical correlation semantics, we extract a local observation window of length *w* around each temporal position:

(4) $$X_{t}^{\mathrm{loc}}\in R^{D\times w}$$

Each variable in the local window is independently standardized as

(5) $${\tilde{X}}_{t}^{\mathrm{loc}}=\mathrm{Std}\left(X_{t}^{\mathrm{loc}}\right)$$

where Std(·) subtracts the local mean and divides by the local standard deviation of each variable. A small positive constant ϵ is added to the standard deviation for numerical stability. The local standardized correlation matrix is then calculated as

(6) $$R_{t}^{\mathrm{loc}}=\frac{1}{w-1}{\tilde{X}}_{t}^{\mathrm{loc}}{\left({\tilde{X}}_{t}^{\mathrm{loc}}\right)}^{T}\in {\left[-1,1\right]}^{D\times D}$$

The element $R_{t,ij}^{\mathrm{loc}}$ represents the local linear correlation between variables *i* and *j*. Its sign therefore provides an explicit statistical interpretation of positive and negative inter-variable correlations. Although the local standardized correlation matrix provides statistically meaningful relation polarity, not every observed correlation contributes equally to forecasting under the current temporal context. We therefore construct a context-dependent attention gate. Specifically, the contextual representations are projected into a shared latent space:

(7) $$E_{t}=Z_{t}W_{E}\in R^{D\times D_{a}}$$

where $W_{E}\in R^{D_{c}\times D_{a}}$ is a learnable projection matrix and $D_{a}$ denotes the latent dimension. The context-dependent attention gate is calculated as

(8) $$G_{t}=\sigma \left(\frac{E_{t}E_{t}^{T}}{\sqrt{D_{a}}}\right)\in {\left(0,1\right)}^{D\times D}$$

where σ(·) denotes the sigmoid function. The shared projection ensures that $G_{t}$ is symmetric, while its elements indicate the context-dependent relevance of different variable pairs. Finally, the dynamic correlation matrix is obtained by combining the local correlation prior with the context-dependent attention gate:

(9) $$S_{t}=R_{t}^{\mathrm{loc}}⊙G_{t}\in {\left[-1,1\right]}^{D\times D}$$

where ⊙ denotes element-wise multiplication. Since $G_{t}$ is strictly non-negative, it adaptively adjusts the contribution of each local correlation without reversing its statistically determined polarity. Moreover, both $R_{t}^{\mathrm{loc}}$ and $G_{t}$ vary across temporal positions, allowing $S_{t}$ to capture temporal changes in correlation strength and polarity. Accordingly, each variable pair (i,j) is associated with a time-varying relation sequence

(10) $$S_{ij}=\left\{S_{1,ij},S_{2,ij},\ldots ,S_{L,ij}\right\}$$

These dynamic correlation weights are subsequently refined through polarity-aware correlation calibration and adaptive sparse relation selection.

### 3.4. Heterogeneous Correlation Module

**Figure 3:**
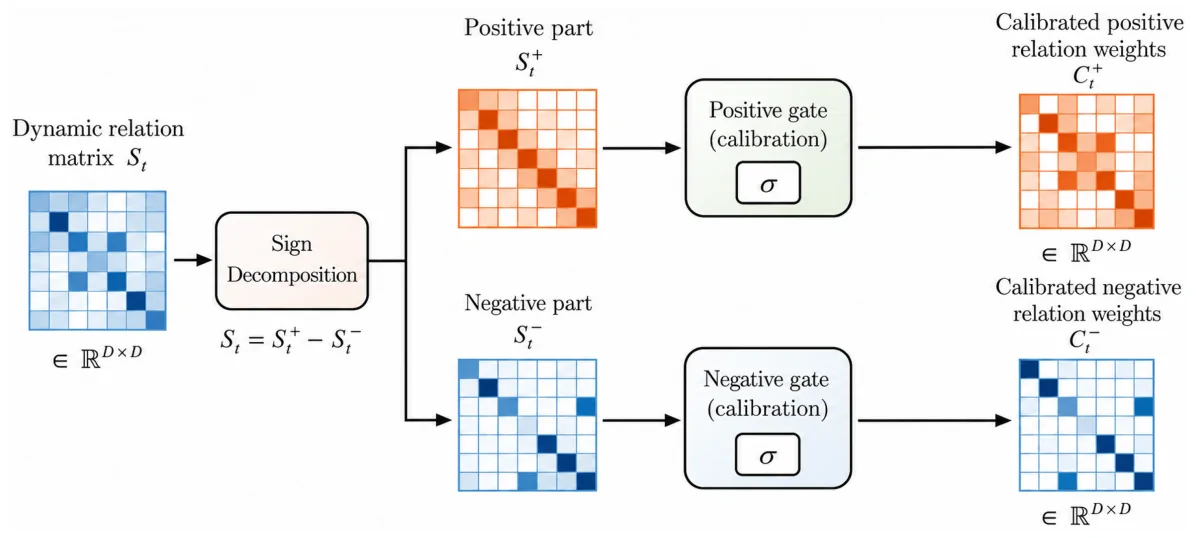
The structure of the Heterogeneous Correlation Module.

DCorr captures the temporal variation in inter-variable correlation strength, but does not explicitly distinguish the heterogeneous effects associated with different relation types. Existing studies have shown that separating positive and negative correlations is beneficial for characterizing heterogeneous relational semantics. Motivated by signed relation modeling [11], we further introduce HCorr on top of the dynamic correlation matrix produced by DCorr. As illustrated in Figure 3, HCorr first separates positive and negative relations and then models them using two independent learnable transformations. In this way, the dynamic correlation information is preserved while different relation types are adaptively calibrated according to their distinct interaction effects.

Given the dynamic correlation matrix $S_{t}\in {\left[-1,1\right]}^{D\times D}$, we first separate its positive and negative components as

(11) $$S_{t}^{+}=\mathrm{ReLU}\left(S_{t}\right),\;S_{t}^{-}=\mathrm{ReLU}\left(-S_{t}\right)$$

Here, $S_{t}^{+}$ and $S_{t}^{-}$ represent the magnitudes of positive and negative inter-variable correlations, respectively. Since both components are derived from the time-varying correlation matrix $S_{t}$, the correlation type of the same variable pair can also vary with temporal context. To model the heterogeneous effects of the two correlation types, separate learnable gates are applied to the positive and negative components:

(12) $$C_{t}^{+}=S_{t}^{+}⊙\sigma \left(w_{+}S_{t}^{+}+b_{+}\right),\;C_{t}^{-}=S_{t}^{-}⊙\sigma \left(w_{-}S_{t}^{-}+b_{-}\right)$$

where $w_{+}$ and $w_{-}$ are learnable scaling parameters, $b_{+}$ and $b_{-}$ are learnable biases, σ(·) denotes the sigmoid function, and ⊙ denotes element-wise multiplication. The two branches are independently parameterized to adaptively calibrate the relation weights associated with positive and negative dependencies. Accordingly, $C_{t}^{+}$ and $C_{t}^{-}$ remain pairwise relation-weight matrices, while explicitly distinguishing the heterogeneous effects of the two relation types. These relation weights are subsequently passed to SCorr for selective retention.

### 3.5. Selective Correlation Modeling

Although DCorr captures temporal variations in inter-variable relations and HCorr distinguishes their heterogeneous types, retaining all pairwise dependencies may still introduce redundant interactions. For example, a traffic sensor may interact with multiple neighboring sensors during congestion propagation, while only a few local dependencies remain informative under free-flow conditions. Therefore, SCorr further characterizes the selectivity of inter-variable dependencies by identifying informative relations from the dynamically estimated and heterogeneously calibrated relation weights.

**Figure 4:**
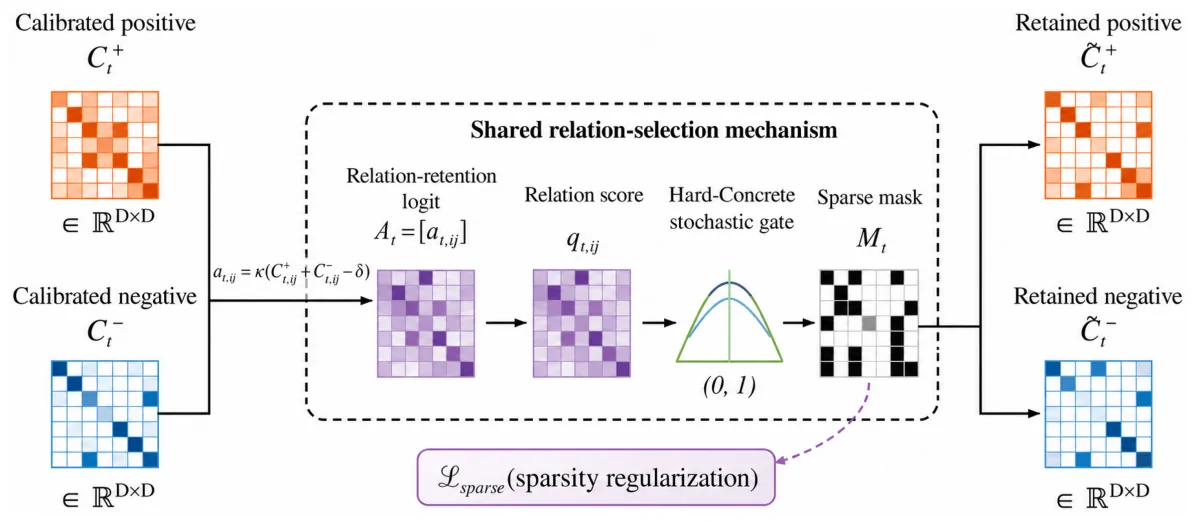
Architecture of the Selective Correlation Module.

Existing relation-selection methods commonly retain the Top-*K* strongest inter-variable relations to suppress redundant interactions. However, this strategy requires a predefined sparsity level and enforces a fixed number of retained relations across different temporal contexts. Such a fixed selection scheme presents a notable limitation for dynamic multivariate time series, where the number of informative inter-variable dependencies may vary with the underlying temporal state. To address this limitation, we introduce an $L_{0}$-regularized stochastic gating mechanism based on the Hard-Concrete relaxation [38], allowing the model to adaptively determine the number of retained relations through learned sparse gates, as illustrated in Figure 4.

Given the heterogeneous relation weights obtained from HCorr, the polarity-independent strength of the relation between variables *i* and *j* is represented by $C_{t,ij}^{+}+C_{t,ij}^{-}$. The tendency to retain the corresponding relation is parameterized as

(13) $$a_{t,ij}=\kappa \left(C_{t,ij}^{+}+C_{t,ij}^{-}-\delta \right)$$

where δ is a learnable relation threshold and κ controls the sharpness of the selection boundary. Since positive and negative relations have already been separately calibrated by HCorr, $C_{t,ij}^{+}+C_{t,ij}^{-}$ provides a polarity-independent measure of the calibrated relation strength. In this way, relation selection is decoupled from relation type.

Following the Hard-Concrete relaxation for differentiable $L_{0}$ regularization [38], we associate each pairwise relation with a stochastic gate. For the relation between the *i*-th and *j*-th variables at time step *t*, a relaxed gate is sampled as

(14) $$r_{t,ij}=\sigma \left(\frac{\log u_{t,ij}-\log\left(1-u_{t,ij}\right)+a_{t,ij}}{\beta }\right),\;u_{t,ij}∼U\left(0,1\right)$$

where σ(·) denotes the sigmoid function, $a_{t,ij}\in R$ is the learnable log-location parameter of the corresponding relation gate, and β>0 is the temperature parameter controlling the smoothness of the continuous relaxation. A smaller β produces a sharper approximation to a binary gate. The sampled gate is subsequently stretched to the interval (γ,ζ) and clipped to [0,1]:

(15) $$M_{t,ij}=\min\left(1,\max\left(0,r_{t,ij}\left(\zeta -\gamma \right)+\gamma \right)\right)$$

where γ<0 and ζ>1 denote the lower and upper bounds of the stretching interval, respectively. The clipping operation allows the resulting gate to attain exact zeros and ones while retaining differentiability over its continuous region. Collecting all pairwise gates yields the relation-selection mask.

(16) $$M_{t}={\left[M_{t,ij}\right]}_{i,j=1}^{D}\in {\left[0,1\right]}^{D\times D}$$

When $M_{t,ij}=0$ the corresponding inter-variable relation is excluded from subsequent information propagation, whereas a larger $M_{t,ij}$ indicates a stronger tendency to retain that relation. The learned selection mask is then applied to the heterogeneous relation weights obtained from HCorr:

(17) $${\tilde{C}}_{t}^{+}=M_{t}⊙C_{t}^{+},\;{\tilde{C}}_{t}^{-}=M_{t}⊙C_{t}^{-}$$

The same selection mask is shared by the two relation types because each signed entry $S_{t,ij}$ is decomposed by ReLU, so $S_{t,ij}^{+}S_{t,ij}^{-}=0$ and at most one polarity branch is active for a given (t,i,j). The subsequent HCorr gates calibrate the active branch but do not remove this mutual exclusivity. Thus, the mask represents one decision—whether the variable-pair relation exists—whereas HCorr represents how its positive or negative effect is calibrated. To encourage selective relation utilization, the expected number of active inter-variable relations is regularized as

(18) $$L_{\mathrm{sparse}}=\frac{1}{LD(D-1)}\sum_{t=1}^{L}\sum_{i\neq j}\sigma \left(a_{t,ij}-\beta \log\frac{-\gamma }{\zeta }\right)$$

Through this formulation, SCorr selectively filters the heterogeneous relation weights obtained from HCorr. The resulting ${\tilde{C}}_{t}^{+}$ and ${\tilde{C}}_{t}^{-}$ represent the retained positive and negative pairwise relation weights, which are subsequently used to guide cross-variable feature aggregation. Following the parameter settings used in the original Hard-Concrete study, we set β=2/3, γ=−0.1, and ζ=1.1 throughout all experiments.

### 3.6. Correlation-Guided Variable Aggregation

**Figure 5:**
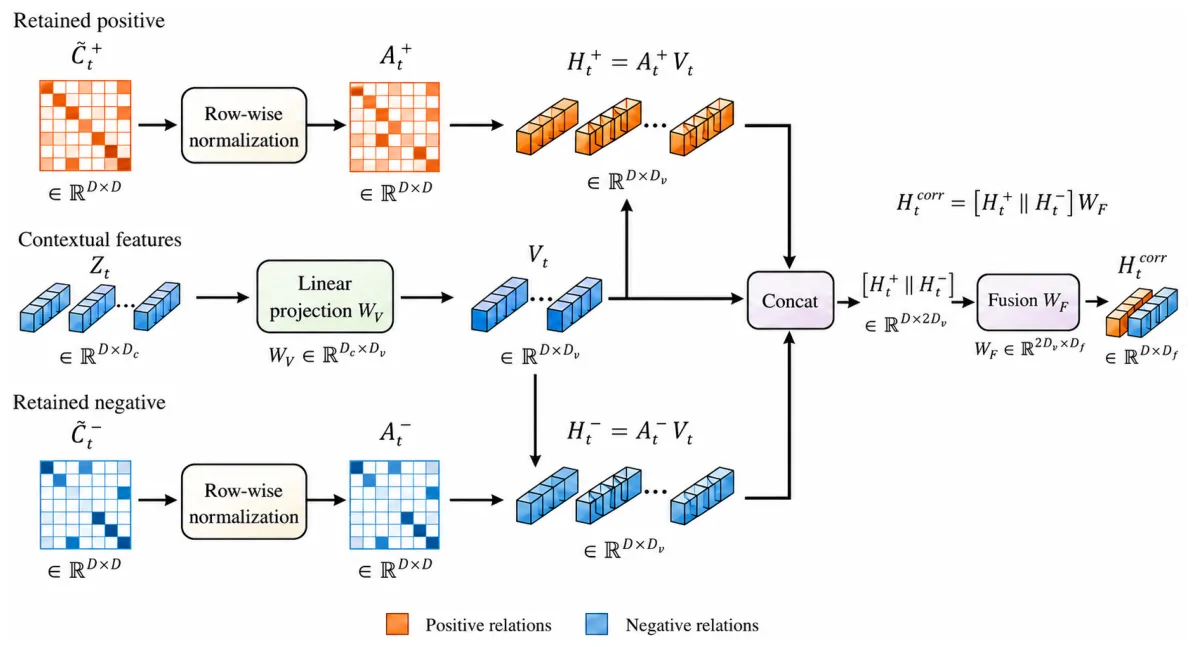
The process of Correlation-guided Variable Aggregation.

The preceding DCorr, HCorr, and SCorr modules operate on inter-variable relations and progressively produce dynamic, heterogeneous, and selectively retained pairwise relation weights. Their outputs therefore specify the strength of information exchange between variable pairs rather than the variable representations themselves. To transform these learned relation weights into correlation-guided feature representations, we use them to aggregate the contextual variable features $Z_{t}$ at each temporal position, as illustrated in Figure 5.

Given the retained positive and negative relation weights ${\tilde{C}}_{t}^{+}$ and ${\tilde{C}}_{t}^{-}$, we first normalize them independently as

(19) $$A_{t,ij}^{+}=\frac{{\tilde{C}}_{t,ij}^{+}}{\sum_{k=1}^{D}{\tilde{C}}_{t,ik}^{+}+\epsilon },\;A_{t,ij}^{-}=\frac{{\tilde{C}}_{t,ij}^{-}}{\sum_{k=1}^{D}{\tilde{C}}_{t,ik}^{-}+\epsilon }$$

where ϵ is a small constant introduced for numerical stability. The resulting matrices $A_{t}^{+}$ and $A_{t}^{-}$ represent the normalized positive and negative relation weights used for cross-variable information propagation. The contextual variable representation $Z_{t}$ is then projected into the feature space used for inter-variable aggregation:

(20) $$V_{t}=Z_{t}W_{V}$$

where $W_{V}$ is a learnable projection matrix. Guided by the learned relation weights, information from different variables is aggregated through the positive and negative dependencies as

(21) $$H_{t}^{+}=A_{t}^{+}V_{t},\;H_{t}^{-}=A_{t}^{-}V_{t}$$

The two relation-guided aggregation results are then combined to obtain the correlation-guided variable representation:

(22) $$H_{t}^{\mathrm{corr}}=\left[H_{t}^{+}\|H_{t}^{-}\right]W_{F}$$

where ‖ denotes feature concatenation and $W_{F}$ is a learnable projection matrix. Applying the above aggregation to all temporal positions yields

(23) $$H^{\mathrm{corr}}=\left\{H_{1}^{\mathrm{corr}},H_{2}^{\mathrm{corr}},\ldots ,H_{L}^{\mathrm{corr}}\right\}$$

which provides the correlation-guided variable representations after information propagation under the learned dynamic, heterogeneous, and selectively retained inter-variable relation weights.

### 3.7. Forecaster

After correlation-guided variable aggregation, the resulting representation $H^{\mathrm{corr}}$ encodes the contextual features of each variable under the learned inter-variable relation weights. To generate the final multi-step prediction, the representations over the look-back window are flattened along the temporal and feature dimensions:

(24) $$H^{\mathrm{flat}}=\mathrm{Flatten}\left(H^{\mathrm{corr}}\right)\in R^{D\times (LD_{h})}$$

where $D_{h}$ denotes the feature dimension of the correlation-guided representation. A linear prediction head is then applied independently to each variable:

(25) $$\hat{Y}=H^{\mathrm{flat}}W_{P}+b_{P}\in R^{D\times H}$$

where $W_{P}\in R^{(LD_{h})\times H}$ and $b_{P}\in R^{H}$ are learnable parameters, and *H* denotes the forecasting horizon. The overall training loss is defined as

(26) $$L=L_{\mathrm{forecast}}+\lambda_{s}L_{\mathrm{sparse}}$$

where $\lambda_{s}$ controls the contribution of the relation-selection regularization.

## 4. Experiments

### 4.1. Experiment Configuration

**Dataset Description**: We evaluate the performance of TimeCorr on eight widely used benchmark datasets. These datasets cover various domains, including electricity (ETTh1, ETTh2, ETTm1, ETTm2, and Electricity), environment (Weather), transportation (Traffic), and finance (Exchange). All of them have been widely used in time-series forecasting tasks. Dataset statistics, including sampling frequency, number of time steps, and number of variables, are summarized in Table 1.

**Implementation Details**: All experiments are conducted on a single NVIDIA GeForce RTX 4080 GPU with 16 GB of memory. Following iTransformer [26], all datasets are partitioned into training, validation, and test sets using the same chronological splitting protocol. All models are optimized using the Adam optimizer. Consistent with established evaluation protocols, forecasting performance is evaluated under four prediction horizons, i.e., H∈{96,192,336,720}. The principal hyperparameter settings of TimeCorr are summarized in Table 2. In the table, “Traffic” and “ECL” denote the corresponding datasets, whereas “others” collectively denotes all remaining datasets. The look-back window length *L* follows the configuration adopted by iTransformer, while the remaining hyperparameters are determined through experimental tuning. Mean squared error (MSE) and mean absolute error (MAE) are adopted as evaluation metrics:

(27) $$\mathrm{MSE}=\frac{1}{N}\sum_{i=1}^{N}{\left(y_{i}-{\hat{y}}_{i}\right)}^{2},$$

(28) $$\mathrm{MAE}=\frac{1}{N}\sum_{i=1}^{N}\left|y_{i}-{\hat{y}}_{i}\right|,$$

where $y_{i}$ and ${\hat{y}}_{i}$ denote the ground-truth and predicted values of the *i*-th observation, respectively, and *N* denotes the total number of predicted scalar values. Lower MSE and MAE values indicate better forecasting performance.

### 4.2. Comparison with State-of-the-Art Models

**Baselines.** We select eight representative forecasting methods as baselines. Five of them explicitly exploit both temporal and inter-variable dependencies through integrated modeling strategies: SageFormer [15], TimeMixier [39], CrossGNN [11], iTransformer [26], and Crossformer [4]. The remaining three methods are temporal-centric forecasting baselines without a dedicated variable-wise dependency-learning mechanism. Specifically, PatchTST [40] adopts a channel-independent architecture, whereas Autoformer [41] and Informer [42] primarily capture dependencies along the temporal dimension without explicitly constructing inter-variable dependency structures.

The experimental results are summarized in Table 3, where lower MSE and MAE values indicate better forecasting performance. Overall, TimeCorr achieves consistently competitive results across nearly all datasets and prediction horizons. Compared with the channel-independent method PatchTST, TimeCorr reduces the average MSE and MAE by 7.59% and 4.32%, respectively. Relative to iTransformer, which explicitly models cross-variable dependencies, the corresponding reductions are 4.64% and 2.74%. These results indicate that a more comprehensive characterization of inter-variable dependencies can effectively improve multivariate time-series forecasting performance. The advantage of TimeCorr is evident on the high-dimensional Electricity and Traffic datasets. On Electricity, TimeCorr reduces the average MSE by 3.57% relative to SageFormer, the strongest competing baseline in terms of MSE, and reduces the average MAE by 1.95% relative to iTransformer, which achieves the best baseline MAE. On Traffic, compared with iTransformer, TimeCorr achieves reductions of 1.99% in MSE and 3.09% in MAE. These results suggest that richer representations of inter-variable dependencies are particularly beneficial when the number of variables is large and the dependency structure becomes more complex. However, TimeCorr performs slightly worse than TimeMixer on the Weather dataset. One possible explanation is that Weather exhibits relatively strong periodicity and multi-scale temporal patterns, which can be more directly exploited by the multi-scale temporal decomposition and mixing mechanisms of TimeMiXer. More importantly, TimeCorr consistently outperforms representative inter-variable dependency modeling methods, including Crossformer, CrossGNN, and SageFormer, which, respectively, focus on the dynamics, heterogeneity, and selectivity of variable relations. Specifically, compared with the more competitive CrossGNN and SageFormer, TimeCorr reduces the average MSE by 6.87% and 1.99%, respectively, with corresponding MAE reductions of 3.8% and 1.52%. Overall, these results demonstrate the effectiveness of jointly characterizing multiple properties of variable relations.

Table 4 further quantifies the variability across the five independent runs. Across the 32 dataset–horizon settings, the standard deviations of TimeCorr range from 0.001 to 0.012 for MSE and from 0.001 to 0.007 for MAE; 31 of the 32 MSE standard deviations are no greater than 0.006. On the high-dimensional Electricity dataset, the MSE and MAE standard deviations are only 0.001–0.002 and 0.001–0.003, respectively, while on Traffic they remain within 0.004–0.006 and 0.002–0.003. These results indicate that the strong mean performance of TimeCorr on the two high-dimensional datasets is reproducible across random initializations rather than being driven by a single favorable run. The largest variability occurs on Exchange at the longest horizon (H=720), where the MSE and MAE standard deviations reach 0.012 and 0.007, respectively, suggesting that long-horizon forecasting on this comparatively small financial dataset is more sensitive to random initialization. Overall, the standard deviations of TimeCorr are comparable to those of the strongest baselines.

**Figure 6:**
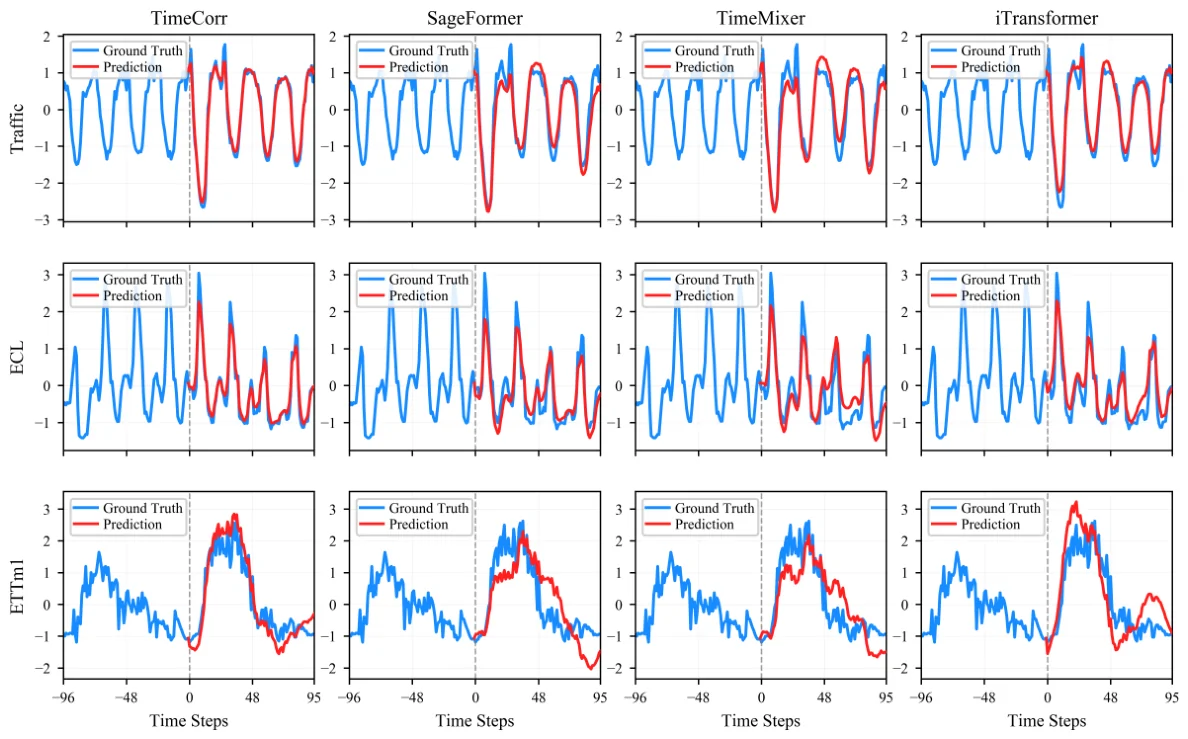
Prediction results of different models on the Traffic, ECL, and ETTm1 datasets.

Figure 6 shows forecasts from TimeCorr, SageFormer, TimeMixer, and iTransformer on the Traffic, ECL, and ETTm1 datasets, where the first 96 time steps correspond to historical observations and the subsequent 96 time steps constitute the forecasting horizon. Overall, all methods are able to capture the main evolution patterns of the ground-truth sequences, while exhibiting different degrees of prediction error around local extrema, rapid fluctuations, and trend transitions. On the Traffic dataset, TimeCorr more consistently preserves the periodic structure and major peak–valley variations in the ground-truth sequence. On the ECL dataset, although the predictions of all methods exhibit a certain degree of smoothing, TimeCorr still closely tracks the locations of major peaks and the overall fluctuation pattern. For the ETTm1 dataset, TimeCorr also maintains good continuity in tracking the rising, peak, and declining stages of the sequence. In contrast, the other methods exhibit more pronounced amplitude deviations or trend shifts around some local extrema. These results demonstrate that TimeCorr maintains stable forecasting performance across different temporal patterns.

### 4.3. Ablation Study and Training Process Visualization

In this section, we first conduct ablation studies to evaluate the contributions of DCorr, HCorr, and SCorr. We then analyze the training processes of TimeCorr and its ablated variants to examine their convergence and generalization. Finally, we visualize the evolution of inter-variable relation representations to illustrate how dynamic, heterogeneous, and selective dependencies are progressively modeled.

#### 4.3.1. Ablation Study

**Figure 7:**
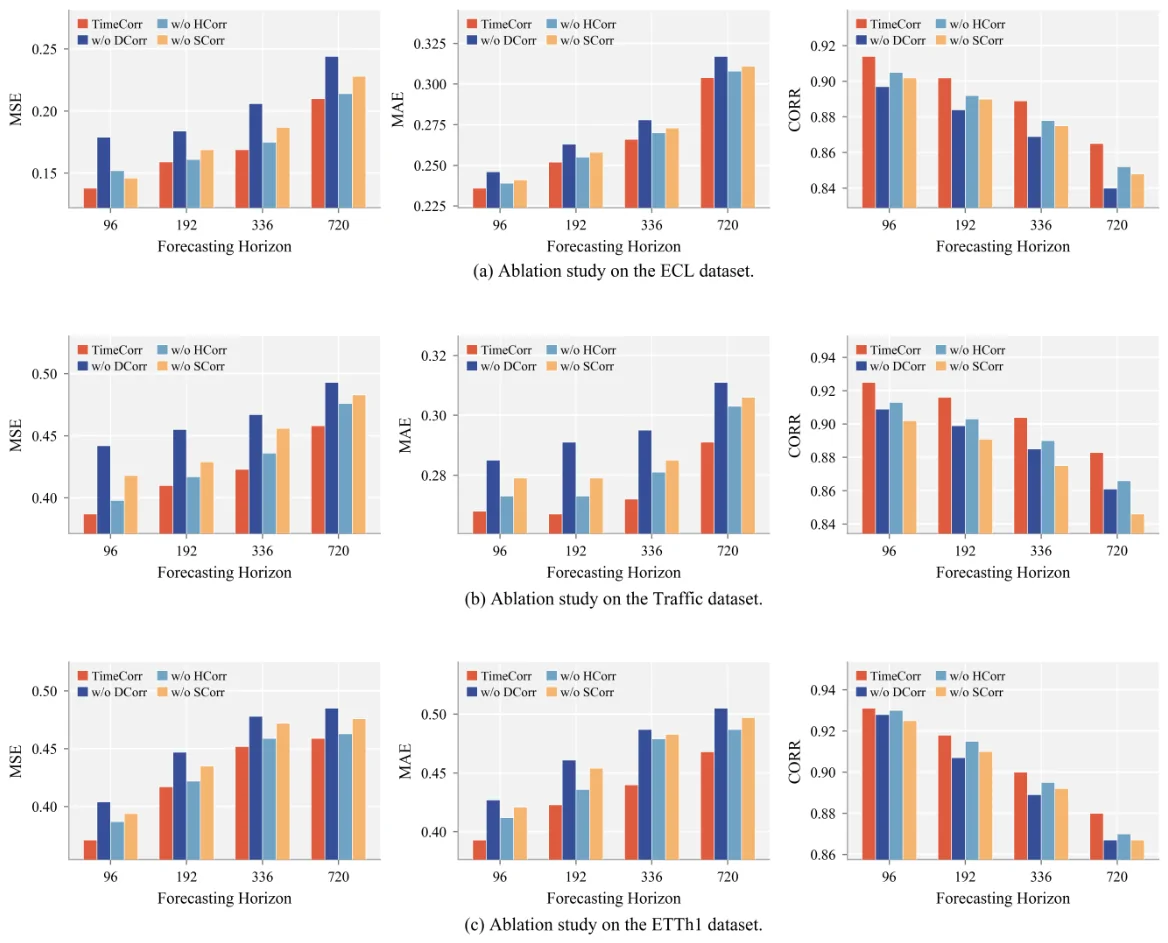
Ablation results of TimeCorr and its variants on the ECL, Traffic, and ETTh1 datasets across different forecasting horizons.

To evaluate the contribution of the three correlation modeling mechanisms in TimeCorr, we conduct ablation studies on the ECL, Traffic, and ETTh1 datasets. Three variants are constructed while keeping all other components and training configurations unchanged. Specifically, (1) ${\mathrm{TimeCorr}}_{w/o\;\mathrm{DCorr}}$ removes the dynamic correlation modeling mechanism by replacing the time-varying relation matrices with a shared static relation matrix over the historical window, thereby preserving cross-variable interaction while eliminating relation dynamics; (2) ${\mathrm{TimeCorr}}_{w/o\;\mathrm{HCorr}}$ removes the heterogeneous correlation modeling mechanism by treating positive and negative inter-variable relations uniformly with a shared propagation transformation; and (3) ${\mathrm{TimeCorr}}_{w/o\;\mathrm{SCorr}}$ removes the selective correlation modeling mechanism by disabling the relation-selection mask, such that all candidate inter-variable relations participate in information propagation. In addition to MSE and MAE, we employ the correlation coefficient (CORR) to measure the consistency between the predicted and ground-truth temporal patterns. Specifically, CORR is defined as

(29) $$\mathrm{CORR}=\frac{1}{N}\sum_{i=1}^{N}\frac{\sum_{t=1}^{H}\left(Y_{i,t}-{\bar{Y}}_{i}\right)\left({\hat{Y}}_{i,t}-{\bar{\hat{Y}}}_{i}\right)}{\sqrt{\sum_{t=1}^{H}{\left(Y_{i,t}-{\bar{Y}}_{i}\right)}^{2}}\sqrt{\sum_{t=1}^{H}{\left({\hat{Y}}_{i,t}-{\bar{\hat{Y}}}_{i}\right)}^{2}}},$$

where *N* denotes the number of variables, *H* is the forecasting horizon, and $Y_{i,t}$ and ${\hat{Y}}_{i,t}$ denote the ground-truth and predicted values of the *i*-th variable at the *t*-th forecasting step, respectively. ${\bar{Y}}_{i}$ and ${\bar{\hat{Y}}}_{i}$ represent the mean values of the corresponding ground-truth and predicted sequences over the forecasting horizon. A lower MSE/MAE and a higher CORR indicate better forecasting performance. The ablation results on ECL, Traffic, and ETTh1 are presented in Figure 7. Several observations can be made. First, the complete TimeCorr consistently achieves lower MSE and MAE and higher CORR across all three datasets and all forecasting horizons, indicating that DCorr, HCorr, and SCorr all contribute positively to the overall forecasting performance.

Removing DCorr produces the most pronounced overall degradation. Compared with the complete model, ${\mathrm{TimeCorr}}_{w/o\;\mathrm{DCorr}}$ increases the average MSE by 20.27%, 10.67%, and 19.13% on ECL, Traffic, and ETTh1, respectively. The corresponding average MAE increases by 4.35%, 7.65%, and 17.05%, while the average CORR decreases from 0.8925 to 0.8725 on ECL, from 0.9070 to 0.8885 on Traffic, and from 0.9100 to 0.8900 on ETTh1. On ETTh1, the MSE gap between the complete model and ${\mathrm{TimeCorr}}_{w/o\;\mathrm{DCorr}}$ further increases from 0.063 at H=96 to 0.106 at H=720. These results indicate that a temporally invariant relation structure is insufficient to characterize the dynamic evolution of inter-variable dependencies across different temporal contexts.

Removing HCorr also results in consistent performance deterioration across all three datasets. ${\mathrm{TimeCorr}}_{w/o\;\mathrm{HCorr}}$ increases the average MSE by 3.85%, 2.92%, and 4.83% on ECL, Traffic, and ETTh1, respectively, and increases the corresponding average MAE by 1.32%, 2.91%, and 5.80%. Meanwhile, the average CORR decreases from 0.8925 to 0.8818 on ECL, from 0.9070 to 0.8930 on Traffic, and from 0.9100 to 0.9025 on ETTh1. This suggests that applying a unified propagation scheme to different types of inter-variable relations weakens the model’s ability to capture heterogeneous dependency patterns, supporting the explicit modeling of heterogeneous correlations in TimeCorr.

Removing SCorr likewise leads to clear performance degradation across all three datasets. Relative to the complete model, ${\mathrm{TimeCorr}}_{w/o\;\mathrm{SCorr}}$ increases the average MSE by 7.99%, 6.44%, and 11.48% on ECL, Traffic, and ETTh1, respectively, while the average MAE increases by 2.36%, 4.64%, and 11.02%. The corresponding average CORR decreases from 0.8925 to 0.8788 on ECL, from 0.9070 to 0.8785 on Traffic, and from 0.9100 to 0.8985 on ETTh1. On the high-dimensional Traffic dataset, the relatively large CORR reduction indicates that indiscriminately propagating information through all candidate variable pairs can introduce redundant dependencies when the relation space is large.

Overall, the results across ETTh1, ECL, and Traffic consistently support the complementary roles of the three modules. DCorr captures temporal variations in inter-variable dependencies, HCorr preserves polarity-specific heterogeneous interaction effects, and SCorr selectively suppresses less informative relations.

We further examine whether positive and negative relations require independent sparse supports. In addition to the default shared-mask configuration, we introduce a separate-mask variant in which two independent Hard-Concrete masks are learned for the positive and negative relation branches. As shown in Table 5, the shared-mask configuration generally provides better and more consistent performance than using separate positive and negative masks. Since HCorr decomposes each signed relation into mutually exclusive positive and negative branches, SCorr only needs to determine whether the corresponding variable-pair interaction should be retained, while HCorr remains responsible for polarity-specific modeling. Therefore, using a shared mask avoids introducing redundant selection variables and provides a simpler and more consistent sparse relation structure.

#### 4.3.2. Training Process Visualization

**Figure 8:**
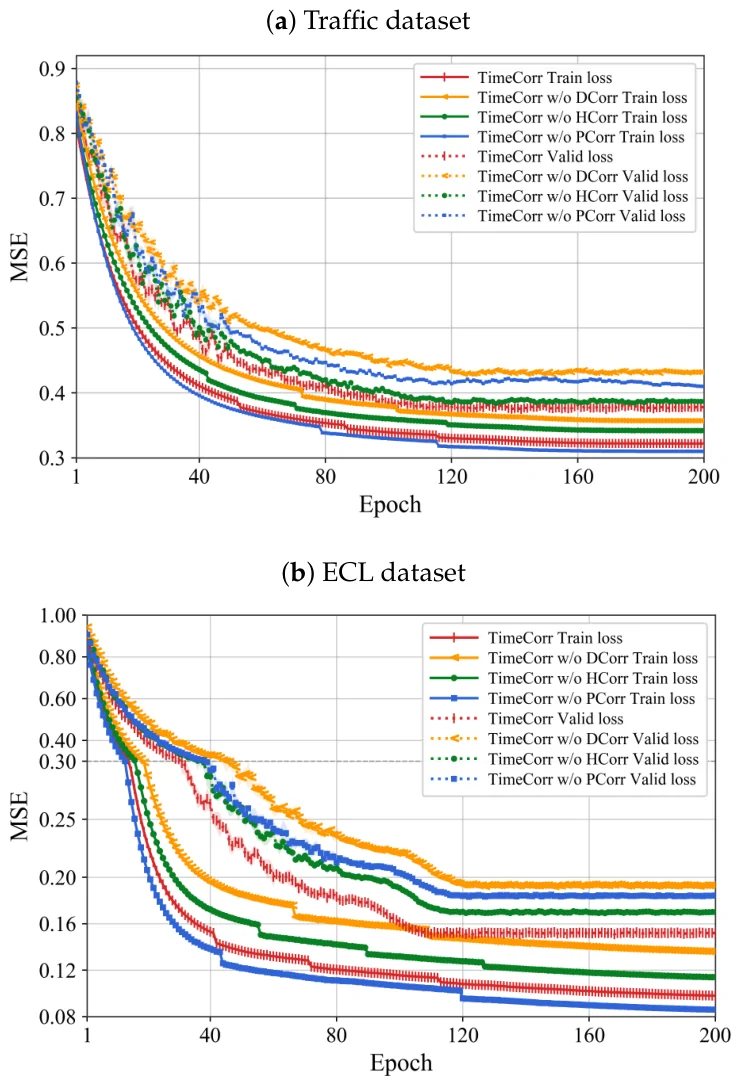
Training and validation MSE curves of TimeCorr and its ablated variants.

We further visualize the training processes of TimeCorr and its three ablated variants, ${\mathrm{TimeCorr}}_{w/o\;\mathrm{DCorr}}$, ${\mathrm{TimeCorr}}_{w/o\;\mathrm{HCorr}}$, and ${\mathrm{TimeCorr}}_{w/o\;\mathrm{SCorr}}$. To reduce the influence of random initialization, each model is trained five times with different random seeds under the same data split, optimizer, learning rate, and other training configurations. The average MSE on the training and validation sets is reported, with the corresponding loss curves shown in Figure 8. ${\mathrm{TimeCorr}}_{w/o\;\mathrm{SCorr}}$ achieves the lowest MSE on the training set but performs worse than TimeCorr on the validation set, indicating a more pronounced overfitting tendency. In contrast, introducing SCorr enables the complete TimeCorr to achieve a lower validation MSE, suggesting that selective relation modeling helps suppress the propagation of redundant dependencies and thereby improves model generalization. Meanwhile, the complete TimeCorr achieves lower MSE on both the training and validation sets than ${\mathrm{TimeCorr}}_{w/o\;\mathrm{DCorr}}$ and ${\mathrm{TimeCorr}}_{w/o\;\mathrm{HCorr}}$, while exhibiting comparable convergence behavior. This indicates that incorporating DCorr and HCorr does not noticeably increase the optimization difficulty, but enables the model to converge to better solutions on the validation set.

**Figure 9:**
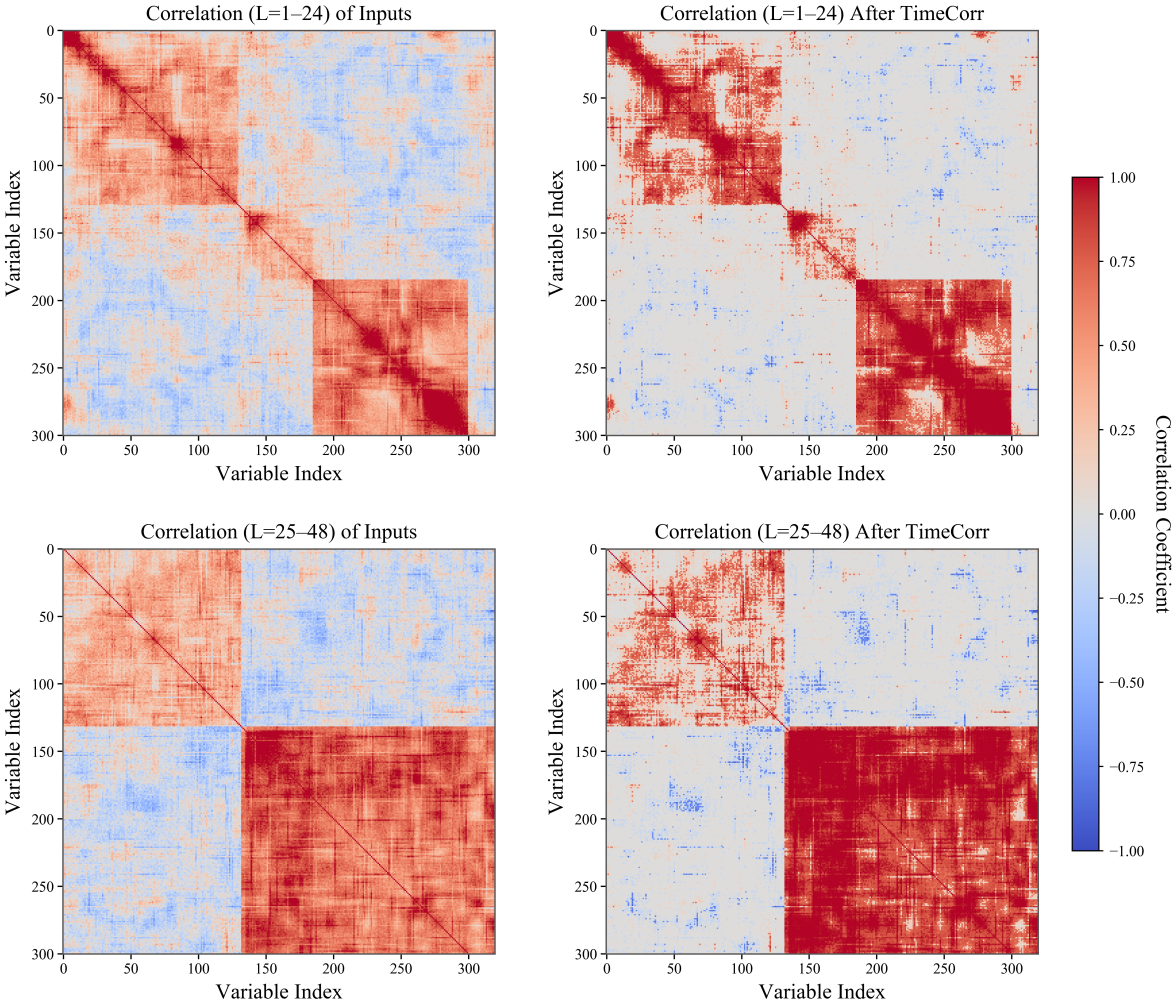
Instance-level visualization of dynamic inter-variable relations on the ECL dataset. The left column shows the empirical Pearson correlation matrices computed from the raw observations, whereas the right column shows the corresponding relation representations learned by TimeCorr.

We conduct an instance-level visualization on the ECL dataset. Since inter-variable correlations cannot be reliably estimated from a single time point, we select two consecutive and equally sized local windows, L=1:24 and L=25:48, from the same test instance. Pearson correlation matrices are computed directly from the raw observations within each window and used as model-independent empirical references. The same input segments are then fed into the complete TimeCorr model, and the final relation representations obtained after DCorr, HCorr, and SCorr are visualized for comparison with the empirical correlation structures. As shown in the left column of Figure 9, the Pearson correlation structures differ substantially between the two local windows. The correlation strengths of several variable pairs change markedly, while some regions, particularly the upper-right and lower-left blocks, exhibit transitions from positive to negative correlation. Correspondingly, the relations learned by TimeCorr exhibit similar changes in the same regions, suggesting that DCorr is capable of adapting the inter-variable relation representation to the evolving input state rather than relying on a fixed dependency structure. In addition, the raw observations contain both pronounced positive and negative correlations, and the corresponding relation patterns are largely preserved in the final relations learned by TimeCorr. This indicates that HCorr can explicitly distinguish inter-variable dependencies with different relation directions. Furthermore, compared with the empirical Pearson correlation matrices, the final relation representations produced by TimeCorr are more concentrated: several weak candidate relations are substantially suppressed, while the dominant positive and negative dependencies are retained. Overall, these results suggest that TimeCorr can simultaneously respond to temporally varying dependency structures, preserve heterogeneous positive and negative relations, and further form a more selective representation of cross-variable dependencies.

The learned SCorr mask also provides a physically interpretable view of the retained cross-variable interactions. Specifically, $M_{t,ij}$ indicates whether the relation from variable *j* to variable *i* is retained during correlation-guided aggregation. Therefore, a consistently retained relation can be directly mapped to the corresponding pair of physical variables and interpreted as a potentially meaningful interaction between them. As shown in Figure 9, SCorr preserves the dominant dependency patterns while suppressing many weak interactions, yielding a sparse relation structure that more clearly highlights the variable pairs contributing to prediction. Accordingly, the learned mask provides a model-level representation for interpreting potentially meaningful relationships among physical variables.

### 4.4. Time Complexity

We analyze the time complexity of the main components of TimeCorr, as summarized in Table 6. Here, *D* denotes the number of variables, *L* the look-back length, *w* the local correlation-window length, *k* the convolution kernel size, $D_{c}$ the contextual feature dimension, $D_{a}$ the shared latent projection dimension, $D_{v}$ the aggregation feature dimension, and *H* the forecasting horizon. The temporal encoder requires $O(DLkD_{c})$. In DCorr, local correlation estimation, shared feature projection, and attention-gate construction require $O(LD^{2}w)$, $O(LDD_{c}D_{a})$, and $O(LD^{2}D_{a})$, respectively. HCorr and SCorr perform element-wise operations on the pairwise relation matrices and therefore both require $O(LD^{2})$. The dominant cost of correlation-guided aggregation is $O(LD^{2}D_{v})$, while the forecasting head requires $O(DLD_{c}H)$. Consequently, the overall time complexity is $O\;\left(LD^{2}\left(w+D_{a}+D_{v}\right)+LDD_{c}\left(k+D_{a}+D_{v}+H\right)\right)$. When the kernel size, local window length, and latent dimensions are treated as fixed, the complexity simplifies to $O(LD^{2}+DLH)$, indicating linear growth with the look-back length and quadratic growth with the number of variables.

Table 7 compares forecasting accuracy at H=96 and computational resource consumption for TimeCorr, TimeMixer, and iTransformer on datasets with different variable dimensions. Overall, TimeCorr improves forecasting accuracy on ETTh1 and Electricity at the cost of moderately increased computational overhead. On ETTh1, TimeCorr achieves the lowest MSE of 0.371, corresponding to reductions of 4.87% and 3.89% relative to TimeMixer (0.390) and iTransformer (0.386), respectively. On Weather, TimeMixer obtains the lowest MSE of 0.157. TimeCorr achieves a comparable MSE of 0.160, which is 1.91% higher than that of TimeMixer but 8.05% lower than that of iTransformer (0.174). On Electricity, TimeCorr achieves the lowest MSE of 0.138, reducing the error by 16.36% and 6.76% relative to TimeMixer (0.165) and iTransformer (0.148), respectively. In terms of computational cost, TimeCorr contains 6.45 M trainable parameters, compared with 3.68 M for TimeMixer and 4.83 M for iTransformer. Its training time, inference latency, and peak GPU memory consumption are also consistently higher because TimeCorr explicitly constructs and processes position-specific cross-variable relation matrices through DCorr, HCorr, and SCorr. As the number of variables increases from 7 on ETTh1 to 321 on Electricity, the resource consumption of all three methods increases, while their parameter counts remain unchanged because the model parameters are shared across variables. For TimeCorr, this increase is particularly associated with the pairwise correlation operations whose computational and memory costs depend on the variable dimension. Nevertheless, the Electricity results indicate that the additional computational cost is accompanied by a larger forecasting improvement in the high-dimensional setting. These results demonstrate that TimeCorr provides a favorable accuracy–computation trade-off when richer cross-variable dependency modeling is beneficial, although lightweight models such as TimeMixer remain more computationally efficient.

### 4.5. Hyperparameter Sensitivity Analysis

**Figure 10:**
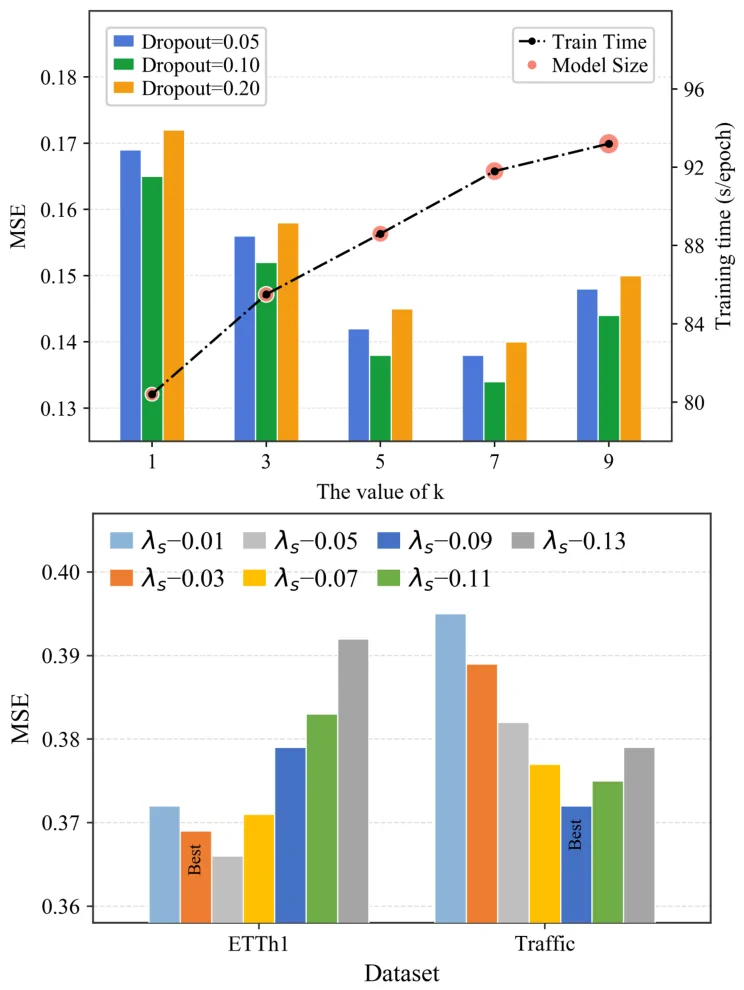
Hyperparameter sensitivity analysis of TimeCorr. (**Top**) effects of the temporal convolution kernel size *k* on MSE and computational cost on the Electricity dataset. (**Bottom**) effects of the sparsity regularization coefficient $\lambda_{s}$ on MSE for the ETTh1 and Traffic datasets.

In this section, we analyze the sensitivity of TimeCorr to two key hyperparameters: the temporal convolution kernel size *k* in DCorr and the sparsity regularization coefficient $\lambda_{s}$ in SCorr. For *k*, we evaluate TimeCorr on Electricity with k∈{1,3,5,7,9} and record both MSE and computational cost to examine the trade-off between forecasting accuracy and efficiency. For $\lambda_{s}$, we conduct experiments on ETTh1 and Traffic, which represent low- and high-dimensional forecasting settings, respectively, to investigate how the appropriate regularization strength varies with variable dimensionality. All remaining hyperparameters are fixed to their default settings. The corresponding results are presented in Figure 10.

**(1) Temporal convolution kernel size *k*:** When k=1, the contextual representation at each temporal position is derived solely from the current observation, without explicitly incorporating information from neighboring positions. Increasing *k* enlarges the local temporal receptive field and provides richer contextual information for estimating dynamic inter-variable correlations, thereby improving forecasting accuracy. However, the reduction in MSE becomes marginal when *k* exceeds 5, whereas the computational cost continues to increase with the kernel size. Therefore, k=5 achieves a favorable balance between forecasting accuracy and computational efficiency and is adopted as the default setting.

**(2) Sparsity regularization coefficient $\lambda_{s}$:** The results exhibit different sensitivity patterns on ETTh1 and Traffic. On the low-dimensional ETTh1 dataset, weak or moderate sparsity regularization is sufficient to suppress redundant interactions, whereas an excessively large $\lambda_{s}$ may remove informative dependencies. In contrast, the high-dimensional Traffic dataset benefits from stronger sparsity regularization because its substantially larger number of candidate variable pairs increases the likelihood of redundant and noisy interactions. This contrast indicates that the appropriate value of $\lambda_{s}$ depends on variable dimensionality: relatively weak regularization is preferable for low-dimensional datasets, whereas stronger regularization is more suitable for high-dimensional datasets. $\lambda_{s}$ is set to 0.07 as the default value in other experiments.

**(3) Local correlation-window length *w*:** To investigate the sensitivity of TimeCorr to the local correlation-window length *w*, we conduct two complementary experiments to examine forecasting accuracy, computational cost, correlation stability, and temporal responsiveness. First, TimeCorr is evaluated on ETTh1, Weather, and Electricity using w∈{4,8,12,24,48}, with the forecasting horizon fixed to H=96. All other model configurations and training settings are kept unchanged. For each value of *w*, we report the test MSE and the inference latency per sample. The inference latency is measured under the same hardware and batch-size configuration after a fixed number of warm-up iterations.

**Figure 11:**
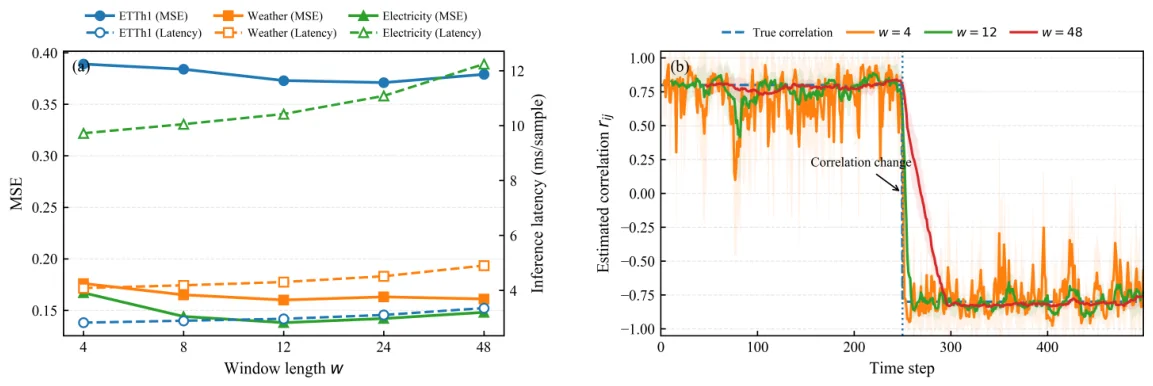
Sensitivity analysis of the local correlation-window length *w*. (**a**) Forecasting MSE and inference latency on ETTh1, Weather, and Electricity. (**b**) Temporal response of the estimated correlation under different window lengths. The light-colored trajectories show the individual realizations, whereas the darker solid curves show their mean.

As shown in Figure 11a, the forecasting error generally decreases as *w* increases from 4 to a moderate value, while further enlarging the window provides no consistent improvement. Specifically, ETTh1 achieves its lowest MSE of 0.371 at w=24, whereas Weather and Electricity obtain their lowest MSE values of 0.160 and 0.138, respectively, at w=12. When *w* is further increased to 48, the MSE increases to 0.379 on ETTh1 and 0.148 on Electricity, while Weather shows only a marginal change from 0.160 to 0.161. These results suggest that a relatively small window may provide insufficient temporal context for stable local correlation estimation, whereas an excessively large window can smooth short-term changes in cross-variable dependencies without yielding additional forecasting benefits. Meanwhile, the inference latency increases consistently with *w* on all three datasets, and this increase is particularly evident on the higher-dimensional Electricity dataset. Therefore, enlarging the local correlation window introduces additional computational cost without necessarily improving forecasting accuracy.

To further isolate the effect of *w* on correlation stability and temporal responsiveness, we construct a controlled correlation-change experiment. Two standardized sequences of length T=500 are generated according to $y_{t}=\rho_{t}x_{t}+\sqrt{1-\rho_{t}^{2}}\epsilon_{t}$, where $x_{t},\epsilon_{t}∼N\left(0,1\right)$. The underlying correlation is set to $\rho_{t}=0.8$ for t<250 and $\rho_{t}=-0.8$ for t≥250, producing an abrupt change from strong positive to strong negative dependence at t=250. The experiment is repeated over five independent realizations, and the mean estimated correlation trajectories are reported.

As illustrated in Figure 11b, the window length directly controls the trade-off between correlation stability and temporal responsiveness. With w=4, the estimated correlation reacts rapidly to the abrupt dependency change, but exhibits substantial short-term fluctuations before and after the change point. Increasing the window to w=12 considerably suppresses these fluctuations while preserving a relatively rapid response to the change. In contrast, w=48 produces the smoothest correlation trajectory but introduces a clearly larger response delay after t=250. Thus, a smaller *w* improves temporal responsiveness at the expense of correlation stability, whereas a larger *w* produces more stable estimates but increases both response delay and computational cost. Considering the forecasting results across the three datasets together with the stability–responsiveness and accuracy–efficiency trade-offs, w=12 provides a suitable overall compromise and is therefore adopted in the main experiments.

### 4.6. Increasing Look-Back Window

**Figure 12:**
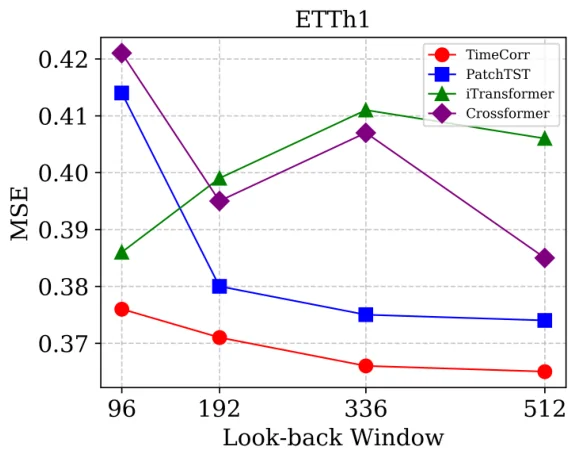
Effect of the look-back window length *L* on the forecasting performance of TimeCorr and baseline models on ETTh1.

Previous work [43] indicates that many attention-based models, including Informer and Autoformer, struggle to achieve higher forecasting accuracy by simply increasing the look-back window length. Nevertheless, a longer look-back window provides a broader historical context that may contain useful predictive information. To examine whether TimeCorr can effectively exploit this additional information, we compare it with the baseline models on ETTh1, using four look-back window lengths, L∈{96,192,336,512}. As shown in Figure 12, TimeCorr achieves the lowest MSE across all evaluated look-back window lengths. Moreover, its MSE generally decreases as *L* increases, demonstrating its ability to extract useful predictive information from extended historical sequences.

### 4.7. Robustness

Due to measurement errors and sensor abnormalities, practical time-series observations may be affected by continuous random disturbances and sparse impulsive corruption. To evaluate the robustness of TimeCorr under these two representative perturbation settings, we conduct experiments on the ETTh1 test set using additive Gaussian noise and Salt-and-Pepper corruption, while keeping the forecasting targets and trained model parameters unchanged. PatchTST is included as a reference model because previous work reports its strong robustness to input perturbations [15]. For additive Gaussian noise, the perturbed input is defined as $\tilde{X}=X+\epsilon$, where $\epsilon ∼N(0,\sigma^{2})$ and σ∈{0,0.1,0.2,0.3,0.4}. The perturbations are applied in the normalized input space to maintain a consistent noise scale across variables. For Salt-and-Pepper corruption, a corruption density ρ∈{0,0.05,0.10,0.20,0.30} is considered. For each corrupted input, ρ/2 of the positions are randomly selected and replaced by the corresponding variable-wise maximum value observed in the normalized training set, while another disjoint ρ/2 are replaced by the corresponding variable-wise minimum value.

**Figure 13:**
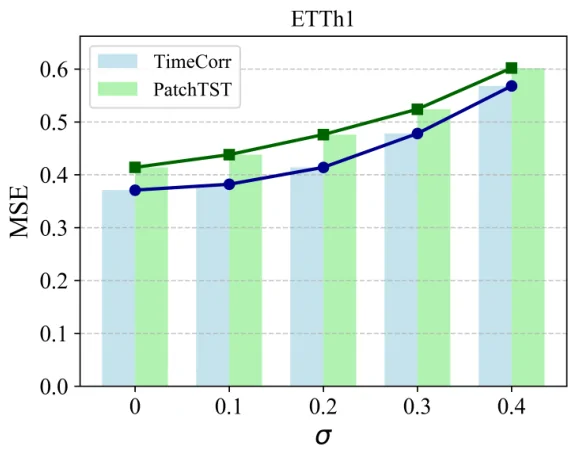
Forecasting MSE of TimeCorr and PatchTST on ETTh1 under different levels of additive Gaussian input noise. The overlaid marker lines connect the same MSE values shown by the bars and emphasize the trend across noise levels.

**Figure 14:**
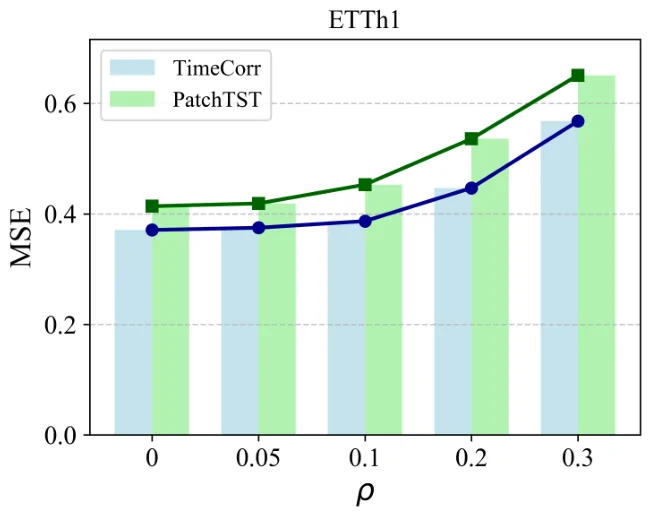
Forecasting MSE of TimeCorr and PatchTST on ETTh1 under different densities of Salt-and-Pepper input noise. Here, ρ denotes the total fraction of corrupted input positions. The overlaid marker lines connect the same MSE values shown by the bars and emphasize the trend across corruption densities.

As shown in Figure 13 and Figure 14, the forecasting errors of both TimeCorr and PatchTST increase as the perturbation intensity becomes stronger under both corruption settings. Under additive Gaussian noise, TimeCorr consistently achieves a lower MSE than PatchTST across all evaluated noise levels. A similar trend is observed under Salt-and-Pepper corruption. The degradation is relatively limited at low corruption densities but becomes more pronounced when ρ increases to 0.20 and 0.30. At ρ=0.30, the MSE of TimeCorr increases from approximately 0.37 to 0.57, whereas that of PatchTST increases from approximately 0.41 to 0.65. TimeCorr therefore maintains a lower forecasting error than PatchTST throughout the tested corruption range, including under relatively severe impulse corruption. Overall, the two experiments indicate that TimeCorr is comparatively less affected than PatchTST by the evaluated continuous Gaussian perturbations and sparse extreme-value corruptions on ETTh1. Nevertheless, both models exhibit increasing errors as the perturbation intensity grows, indicating that strong input corruption remains challenging.

## 5. Conclusions

This paper proposes TimeCorr, a unified inter-variable correlation learning framework for long-term multivariate time series forecasting. TimeCorr jointly characterizes dynamic, heterogeneous, and selective correlations to account for temporal variations in dependency strength, polarity-specific interactions, and the selective occurrence of informative variable relations. The learned correlation structures are subsequently used to guide cross-variable information aggregation for forecasting. Extensive experiments on eight benchmark datasets demonstrate the superior overall performance of TimeCorr, particularly on the high-dimensional Electricity and Traffic datasets. Ablation studies and relation visualizations further confirm the effectiveness of the three correlation modeling mechanisms. Additional experiments show that TimeCorr can benefit from extended historical contexts and achieves lower MSE than PatchTST under the tested additive Gaussian and Salt-and-Pepper perturbations on ETTh1.

From a deployment perspective, TimeCorr has potential for industrial soft-sensing scenarios involving continuously monitored multivariate process data with evolving cross-variable dependencies. Its progressive correlation-learning framework enables DCorr to capture time-varying dependencies, HCorr to distinguish and calibrate positive and negative process couplings, and SCorr to suppress redundant or weak interactions before correlation-guided aggregation. These characteristics may be particularly useful when process measurements contain redundant channel variables that are costly to obtain. However, as a supervised end-to-end forecasting model, TimeCorr does not explicitly address abrupt concept drift, severe sensor faults, or operating regimes outside the training distribution.

Despite these results, TimeCorr still has several limitations. First, constructing position-specific correlation matrices requires evaluating pairwise interactions among all variables at every temporal position. The resulting computational and memory costs increase quadratically with the number of variables, which may restrict its application to extremely high-dimensional time series. In particular, the main scalability bottlenecks lie in the pairwise correlation estimation and attention-gate construction in DCorr, together with the subsequent dense correlation-guided aggregation. Future work will investigate low-rank correlation representations, hierarchical relation selection, and sparse computation to reduce the cost of these components and improve the scalability of TimeCorr for ultra-high-dimensional industrial sensor data. Second, TimeCorr does not explicitly distinguish higher-order, lead-lag, or causal interactions among variables. Extending the framework to incorporate these dependency structures while preserving its progressive correlation-learning mechanism represents another important direction for future research.

## Figures and Tables

**Table 1:** Summary of the multivariate time-series datasets.

Dataset	Domain	Granularity	Variates	Time Steps
ETTh1	Electricity	1 h	7	17,420
ETTh2	Electricity	1 h	7	17,420
ETTm1	Electricity	15 min	7	69,680
ETTm2	Electricity	15 min	7	69,680
Weather	Environment	10 min	21	52,696
Electricity	Electricity	1 h	321	26,304
Traffic	Traffic	1 h	862	17,544
Exchange	Economic	1 day	8	7588

**Table 2:** Hyperparameter settings.

Hyperparameter	Value	Hyperparameter	Value
patience	30	Dropout	0.1
epochs	200	learning rate	0.001
*L*	96	*H*	{96, 192, 336, 720}
$D_{c}$	16	batch size	16 (Traffic&ECL), 64 (others)
$D_{a}$	512	number of attention heads	8

**Table 3:** Mean forecasting results over five independent runs for long-term time-series forecasting.

Models		TimeCorr		SageFormer		TimeMixer		CrossGNN		iTransformer		Crossformer		PatchTST		Autoformer		Informer	
Metric		MSE	MAE	MSE	MAE	MSE	MAE	MSE	MAE	MSE	MAE	MSE	MAE	MSE	MAE	MSE	MAE	MSE	MAE
ETTh1	96	**0.371**	**0.393**	0.377	0.397	0.390	0.411	0.394	0.400	0.386	0.405	0.421	0.448	0.414	0.419	0.449	0.459	0.865	0.713
	192	**0.417**	**0.423**	0.423	0.425	0.442	0.442	0.444	0.429	0.441	0.436	0.534	0.515	0.460	0.445	0.500	0.482	1.008	0.792
	336	**0.452**	**0.440**	0.459	0.445	0.480	0.468	0.481	0.448	0.487	0.458	0.656	0.581	0.501	0.466	0.521	0.496	1.107	0.809
	720	**0.459**	0.468	0.465	**0.466**	0.494	0.488	0.489	0.472	0.503	0.491	0.849	0.709	0.500	0.488	0.514	0.512	1.181	0.865
ETTh2	96	**0.285**	**0.337**	0.286	0.338	0.328	0.371	0.293	0.340	0.297	0.349	1.142	0.772	0.302	0.348	0.346	0.388	3.755	1.525
	192	**0.366**	0.393	0.368	0.394	0.402	0.414	0.377	**0.391**	0.380	0.400	1.784	1.022	0.388	0.400	0.456	0.452	5.602	1.931
	336	**0.409**	**0.425**	0.413	0.429	0.435	0.443	0.446	0.448	0.428	0.432	2.642	1.404	0.426	0.433	0.482	0.486	4.472	1.835
	720	**0.421**	**0.444**	0.429	0.449	0.430	0.451	0.427	0.448	0.431	0.445	3.111	1.501	0.431	0.446	0.515	0.511	3.647	1.625
ETTm1	96	0.322	**0.361**	0.324	0.362	**0.319**	0.366	0.331	0.368	0.334	0.368	0.361	0.402	0.329	0.367	0.505	0.475	0.672	0.571
	192	**0.357**	**0.385**	0.369	0.392	0.376	0.397	0.376	0.391	0.377	0.391	0.403	0.440	0.372	0.389	0.553	0.496	0.795	0.669
	336	**0.398**	**0.401**	0.403	0.408	0.417	0.422	0.411	0.412	0.426	0.420	0.551	0.535	0.407	0.410	0.621	0.537	1.212	0.871
	720	**0.453**	**0.439**	0.457	0.441	0.481	0.458	0.475	0.448	0.491	0.459	0.720	0.649	0.454	0.442	0.671	0.561	1.166	0.823
ETTm2	96	**0.169**	**0.251**	0.175	0.257	0.177	0.262	0.180	0.262	0.180	0.264	0.253	0.347	0.175	0.259	0.255	0.339	0.365	0.453
	192	**0.237**	**0.295**	0.239	0.299	0.247	0.307	0.245	0.304	0.250	0.309	0.421	0.483	0.241	0.302	0.281	0.340	0.533	0.563
	336	**0.291**	**0.329**	0.299	0.338	0.312	0.346	0.306	0.342	0.311	0.348	1.276	0.805	0.305	0.343	0.339	0.372	1.363	0.887
	720	**0.388**	**0.392**	0.395	0.395	0.414	0.403	0.397	0.399	0.412	0.407	3.786	1.354	0.402	0.400	0.433	0.432	3.379	1.338
Weather	96	0.160	0.208	0.162	0.212	**0.157**	**0.206**	0.174	0.216	0.174	0.214	0.167	0.219	0.177	0.218	0.266	0.336	0.300	0.384
	192	**0.206**	0.256	0.211	0.258	0.208	**0.254**	0.227	0.260	0.221	0.258	0.208	0.277	0.225	0.259	0.307	0.367	0.598	0.544
	336	**0.265**	**0.294**	0.271	0.296	0.266	0.295	0.283	0.301	0.278	0.296	0.285	0.320	0.278	0.297	0.359	0.395	0.578	0.523
	720	0.342	0.347	0.345	0.349	**0.339**	**0.342**	0.359	0.352	0.358	0.349	0.388	0.391	0.354	0.362	0.419	0.428	1.059	0.741
Electricity	96	**0.138**	**0.236**	0.147	0.246	0.165	0.274	0.156	0.249	0.148	0.240	0.213	0.300	0.181	0.270	0.201	0.317	0.274	0.368
	192	**0.159**	**0.252**	0.161	0.259	0.184	0.292	0.169	0.260	0.162	0.253	0.290	0.351	0.188	0.274	0.222	0.334	0.296	0.386
	336	**0.169**	**0.266**	0.180	0.279	0.195	0.302	0.186	0.276	0.178	0.269	0.348	0.389	0.204	0.293	0.231	0.338	0.300	0.394
	720	**0.210**	**0.304**	0.213	0.309	0.231	0.332	0.228	0.312	0.225	0.317	0.405	0.425	0.246	0.324	0.254	0.361	0.373	0.439
Traffic	96	**0.387**	**0.268**	0.408	0.271	0.616	0.361	0.465	0.322	0.395	0.272	0.544	0.307	0.462	0.295	0.613	0.388	0.719	0.391
	192	**0.410**	**0.267**	0.421	0.279	0.632	0.371	0.476	0.321	0.417	0.276	0.566	0.315	0.466	0.296	0.616	0.382	0.696	0.379
	336	**0.423**	**0.272**	0.438	0.283	0.649	0.379	0.493	0.327	0.433	0.283	0.571	0.314	0.482	0.304	0.622	0.337	0.777	0.420
	720	**0.458**	**0.291**	0.477	0.308	0.712	0.399	0.524	0.323	0.467	0.302	0.599	0.313	0.514	0.322	0.660	0.408	0.864	0.472
Exchange	96	**0.080**	**0.201**	0.084	0.203	0.102	0.230	0.086	0.204	0.086	0.206	0.256	0.367	0.088	0.205	0.197	0.323	0.847	0.752
	192	**0.175**	0.300	0.177	**0.298**	0.195	0.317	0.185	0.305	0.177	0.299	0.469	0.508	0.176	0.299	0.300	0.369	1.204	0.895
	336	**0.329**	**0.397**	0.338	0.416	0.359	0.436	0.346	0.426	0.338	0.417	0.901	0.741	0.341	0.422	0.509	0.524	1.672	1.036
	720	**0.820**	**0.679**	0.826	0.683	0.940	0.738	0.874	0.704	0.847	0.691	1.398	0.965	0.901	0.714	1.447	0.941	2.478	1.310
1st Count		**55**		2		6		1		0		0		0		0		0	

We compare extensive competitive models under different prediction lengths (96, 192, 336, 720). The input sequence length is set to 96. Bold values indicate the best forecasting result for each dataset, prediction horizon, and evaluation metric.

**Table 4:** Standard deviations of the forecasting results over five independent runs.

Models		TimeCorr		SageFormer		TimeMixer		CrossGNN		iTransformer		Crossformer		PatchTST		Autoformer		Informer	
Metric		Std. (MSE)	Std. (MAE)	Std. (MSE)	Std. (MAE)	Std. (MSE)	Std. (MAE)	Std. (MSE)	Std. (MAE)	Std. (MSE)	Std. (MAE)	Std. (MSE)	Std. (MAE)	Std. (MSE)	Std. (MAE)	Std. (MSE)	Std. (MAE)	Std. (MSE)	Std. (MAE)
ETTh1	96	0.003	0.003	0.004	0.003	0.004	0.003	0.005	0.003	0.004	0.003	0.005	0.004	0.004	0.003	0.004	0.004	0.010	0.005
	192	0.005	0.003	0.005	0.003	0.004	0.004	0.005	0.004	0.004	0.004	0.006	0.004	0.005	0.004	0.006	0.004	0.011	0.008
	336	0.005	0.004	0.006	0.004	0.005	0.004	0.006	0.004	0.005	0.004	0.008	0.005	0.005	0.004	0.007	0.005	0.014	0.007
	720	0.006	0.004	0.006	0.004	0.005	0.005	0.007	0.005	0.006	0.005	0.012	0.007	0.006	0.005	0.007	0.005	0.015	0.010
ETTh2	96	0.003	0.003	0.003	0.003	0.003	0.002	0.004	0.003	0.003	0.002	0.011	0.006	0.003	0.002	0.004	0.003	0.042	0.012
	192	0.004	0.003	0.004	0.003	0.004	0.003	0.005	0.003	0.004	0.003	0.021	0.008	0.004	0.004	0.006	0.004	0.050	0.015
	336	0.004	0.004	0.005	0.004	0.005	0.004	0.006	0.004	0.005	0.004	0.035	0.013	0.005	0.003	0.007	0.005	0.050	0.016
	720	0.005	0.004	0.006	0.004	0.005	0.004	0.006	0.004	0.005	0.005	0.043	0.014	0.005	0.005	0.008	0.005	0.048	0.015
ETTm1	96	0.003	0.002	0.003	0.003	0.003	0.002	0.003	0.003	0.003	0.003	0.004	0.003	0.003	0.003	0.005	0.003	0.006	0.005
	192	0.004	0.003	0.004	0.003	0.004	0.003	0.004	0.003	0.004	0.003	0.004	0.004	0.004	0.003	0.006	0.005	0.009	0.005
	336	0.004	0.003	0.004	0.004	0.004	0.003	0.005	0.004	0.005	0.003	0.006	0.005	0.005	0.003	0.007	0.005	0.016	0.008
	720	0.005	0.004	0.006	0.004	0.005	0.004	0.006	0.005	0.006	0.004	0.008	0.006	0.005	0.004	0.008	0.006	0.016	0.008
ETTm2	96	0.002	0.002	0.002	0.002	0.002	0.002	0.002	0.002	0.002	0.002	0.003	0.002	0.001	0.002	0.003	0.003	0.003	0.004
	192	0.002	0.002	0.002	0.002	0.002	0.002	0.003	0.003	0.003	0.002	0.004	0.004	0.003	0.002	0.003	0.003	0.006	0.005
	336	0.003	0.003	0.003	0.003	0.003	0.003	0.003	0.003	0.004	0.003	0.014	0.008	0.004	0.003	0.004	0.003	0.018	0.008
	720	0.005	0.003	0.004	0.004	0.005	0.003	0.005	0.004	0.005	0.003	0.044	0.014	0.005	0.003	0.005	0.005	0.047	0.013
Weather	96	0.001	0.002	0.002	0.001	0.001	0.001	0.002	0.002	0.002	0.001	0.002	0.002	0.002	0.001	0.002	0.003	0.003	0.003
	192	0.002	0.002	0.002	0.002	0.002	0.002	0.002	0.002	0.002	0.002	0.002	0.002	0.002	0.002	0.003	0.003	0.006	0.005
	336	0.002	0.002	0.003	0.002	0.002	0.002	0.003	0.002	0.003	0.003	0.003	0.003	0.003	0.003	0.004	0.003	0.006	0.004
	720	0.004	0.003	0.004	0.003	0.003	0.003	0.005	0.003	0.004	0.003	0.005	0.003	0.004	0.003	0.005	0.004	0.012	0.007
Electricity	96	0.001	0.001	0.001	0.002	0.001	0.002	0.001	0.002	0.001	0.002	0.002	0.002	0.002	0.002	0.002	0.002	0.003	0.003
	192	0.001	0.002	0.002	0.002	0.002	0.002	0.002	0.002	0.002	0.002	0.003	0.003	0.002	0.002	0.002	0.003	0.003	0.003
	336	0.002	0.002	0.002	0.002	0.002	0.002	0.002	0.002	0.002	0.002	0.004	0.003	0.002	0.002	0.002	0.003	0.004	0.003
	720	0.002	0.003	0.003	0.003	0.002	0.002	0.003	0.003	0.003	0.002	0.004	0.004	0.003	0.003	0.003	0.004	0.005	0.004
Traffic	96	0.004	0.002	0.004	0.002	0.007	0.003	0.005	0.003	0.004	0.002	0.006	0.002	0.005	0.002	0.007	0.003	0.007	0.003
	192	0.005	0.002	0.006	0.003	0.007	0.003	0.005	0.003	0.005	0.002	0.006	0.003	0.005	0.002	0.007	0.004	0.009	0.003
	336	0.005	0.002	0.005	0.003	0.008	0.003	0.006	0.003	0.005	0.002	0.007	0.003	0.006	0.003	0.008	0.004	0.011	0.004
	720	0.006	0.003	0.007	0.003	0.009	0.003	0.007	0.004	0.006	0.003	0.008	0.003	0.007	0.003	0.009	0.005	0.013	0.005
Exchange	96	0.001	0.002	0.001	0.002	0.001	0.002	0.001	0.002	0.001	0.002	0.003	0.003	0.001	0.002	0.003	0.003	0.010	0.007
	192	0.002	0.003	0.002	0.003	0.002	0.003	0.002	0.003	0.002	0.003	0.006	0.005	0.002	0.003	0.004	0.004	0.017	0.009
	336	0.004	0.004	0.004	0.005	0.005	0.004	0.005	0.005	0.005	0.004	0.012	0.008	0.005	0.004	0.007	0.005	0.026	0.011
	720	0.012	0.007	0.011	0.008	0.013	0.007	0.013	0.009	0.013	0.007	0.020	0.012	0.013	0.007	0.022	0.012	0.042	0.015

Standard deviations of MSE and MAE over multiple independent runs under different prediction lengths (96, 192, 336, and 720). The input sequence length is set to 96.

**Table 5:** Comparison between shared and separate SCorr masks on the Electricity and Traffic datasets.

Dataset	Mask Configuration	Avg. MSE	Avg. MAE	Avg. CORR
Electricity	Shared mask (TimeCorr)	**0.169**	**0.265**	**0.899**
	Separate masks	0.174	0.272	0.893
Traffic	Shared mask (TimeCorr)	0.420	**0.275**	**0.907**
	Separate masks	**0.418**	0.279	0.898

Bold values indicate the better result between the shared-mask and separate-mask configurations for each dataset and metric; lower MSE and MAE and higher CORR are better.

**Table 6:** Time complexity of the main components of TimeCorr.

Component	Time Complexity	Simplified Complexity
Temporal Encoder	$O(DLkD_{c})$	O(DL)
DCorr	$O(LD^{2}w+LDD_{c}D_{a}+LD^{2}D_{a})$	$O(LD^{2})$
HCorr	$O(LD^{2})$	$O(LD^{2})$
SCorr	$O(LD^{2})$	$O(LD^{2})$
Correlation-guided Aggregation	$O(LDD_{c}D_{v}+LD^{2}D_{v}+LDD_{v}D_{c})$	$O(LD^{2})$
Forecasting Head	$O(DLD_{c}H)$	O(DLH)
Overall TimeCorr	$O\;\left(LD^{2}\left(w+D_{a}+D_{v}\right)+LDD_{c}\left(k+D_{a}+D_{v}+H\right)\right)$	$O(LD^{2}+DLH)$

**Table 7:** Comparison of forecasting accuracy at H=96 and computational resources of TimeCorr, TimeMixer, and iTransformer on datasets with different variable dimensions.

Dataset	Model	Variables *D*	Params (M)	MSE (H=96)	Training/Epoch (s)	Total Training (min)	Inference (ms/Sample)	Peak Memory (GB)
ETTh1	TimeMixer	7	**3.68**	0.390	**6.1**	**16.2**	**1.8**	**0.46**
	iTransformer	7	4.83	0.386	7.2	19.1	2.3	0.55
	TimeCorr	7	6.45	**0.371**	11.8	24.7	3.1	0.72
Weather	TimeMixer	21	**3.68**	**0.157**	**17.6**	**57.8**	**2.8**	**0.91**
	iTransformer	21	4.83	0.174	20.8	68.3	3.5	1.18
	TimeCorr	21	6.45	0.160	32.6	85.4	4.5	1.52
Electricity	TimeMixer	321	**3.68**	0.165	**58.3**	**143.0**	**6.7**	**1.55**
	iTransformer	321	4.83	0.148	76.5	187.5	9.2	2.20
	TimeCorr	321	6.45	**0.138**	95.8	223.6	11.0	3.05

Bold values indicate the lowest value within each dataset and column.

## Data Availability

Data will be made available upon request.
